# The absolute number of circulating Treg cells is reduced in difficult-to-treat RA patients and is ameliorated by low-dose IL-2

**DOI:** 10.3389/fimmu.2025.1522893

**Published:** 2025-02-06

**Authors:** Huanhuan Yan, Xiaoyu Zi, Huer Yan, Xiaoying Zhang, Jie Bai, Chong Gao, Xiaofeng Li, Caihong Wang

**Affiliations:** ^1^ Department of Rheumatology, The Second Hospital of Shanxi Medical University, Taiyuan, Shanxi, China; ^2^ Department of Rheumatology, Shanxi Key Laboratory of Immunomicroecology, Taiyuan, Shanxi, China; ^3^ Department of Rheumatology, Shanxi Precision Medical Engineering Research Center for Rheumatology, Taiyuan, Shanxi, China; ^4^ College of Basic Medicine, Shanxi Medical University, Taiyuan, Shanxi, China; ^5^ Pathology, Joint Program in Transfusion Medicine, Brigham and Women’s Hospital/Children’s Hospital, Harvard Medical School, Boston, MA, United States

**Keywords:** difficult-to-treat rheumatoid arthritis, regulatory T cells, T helper 17 cells, low-dose interleukin-2, cytokines

## Abstract

**Objective:**

Circulating regulatory T cells (Tregs) are closely related to immune tolerance and maintenance of immune homeostasis. Perhaps, there is a unique immune cell phenotype for difficult-to-treat rheumatoid arthritis (D2T RA). Low-dose interleukin-2 (IL-2) has been considered for the treatment of autoimmune diseases. This study focused on the uniqueness of D2T RA lymphocyte subsets and the feasibility of low-dose IL-2 therapy.

**Methods:**

Participants included 1,042 RA patients who were divided into three groups according to the presence or absence of treatment and their response to treatment in the last 6 months—new group, treated group, and D2T group—and 339 healthy controls (HCs). A total of 381 patients—107, 151, and 123 in each of the three experimental groups—received low-dose IL-2 treatment [0.5 million international units (MIU) per day, subcutaneous injection from day 1 to day 5]. The absolute numbers of peripheral blood lymphocyte subsets were detected by flow cytometry (FCM) and serum cytokine levels were detected by flow cytometry bead array (CBA).

**Results:**

The absolute number of T, CD4^+^ T, and Treg cells in the D2T RA group was lower than that in the HC, new, and treated RA groups. Compared with the HC and new RA group, the ratio of Th17/Treg cells in the D2T RA group increased. The new, treated, and D2T RA groups had higher cytokine levels than the HC. The number of Treg cells in RA patients was negatively correlated with the disease activity index. Treg cells in the new, treated, and D2T RA groups could be increased by low-dose IL-2 therapy without any side effects.

**Conclusions:**

The number of lymphocytes and subsets in D2T RA patients was reduced, especially Treg cells, resulting in a shift in the balance of effector T cells/Treg cells toward effector T cells, which is ameliorated by low-dose IL-2 without obvious side effects.

## 1 Introduction

Rheumatoid arthritis (RA) is a typical chronic autoimmune disease characterized by synovial inflammation and/or joint destruction (1, 2). Although the “treatment-to-target strategy” including disease-modifying antirheumatic drugs (DMARDs) and targeted DMARDs drugs is widely used in clinical practice to strictly control the disease (3), there are still 5%–20% of patients with persistent non-remission (4), which are often referred to as difficult-to-treat (D2T) RA (5). In 2021, the European League Against Rheumatism (EULAR) defined D2T RA as the presence of persistent signs or symptoms following the failure of treatment with at least two biologic agents or DMARD-targeting drugs (6, 7). Although RA is difficult to cure, early diagnosis and timely intervention are very important to slow the development of the disease and prevent irreversible joint destruction.

At present, it has become a consensus that immune tolerance deficiency caused by insufficient number and/or function of regulatory T cells (Tregs) is an important cause of the pathogenesis of RA (8, 9), which opens up new therapeutic possibilities for autoimmune diseases including RA, that is, qualitative and/or quantitative increase of Treg cells (9, 10). EULAR pointed out that D2T RA is a heterogeneous disease in terms of severity, clinical course, and therapeutic response, requiring complex management (6, 11, 12). Moreover, we know that the breakdown of immune tolerance and the production of autoantibodies usually precede clinical disease for several years or even longer (13). Therefore, there is reason to believe that D2T RA may have a distinct immunophenotype. However, until now, there have been no large-scale data to characterize the distinct immunophenotype of D2T RA, which has caused problems for further study.

Interleukin-2 (IL-2) is a soluble T-cell growth factor produced primarily by antigen-activated T cells (14, 15). Low-dose IL-2 can prevent effector T-cell activation and stimulate Treg cell proliferation at the same time, which has been widely used clinically as a treatment for autoimmune diseases (16–18). In addition, Treg cells are more sensitive to IL-2 than effector T cells when the IL-2 dose is low [most experts believe this number to be 0.5–1 million international units (MIU)/day], due to the high expression of CD25 molecules on the surface of Treg cells (17). In 2023, our team analyzed 18 studies around the world on the percentage of Treg cells in patients with rheumatic disease with Ld IL-2 and concluded that Ld IL-2 treatment increases the proportion of Treg cells (9). Ld IL-2, due to its powerful ability to stimulate Treg cells, can largely balance the immune disorder in RA patients (19). We think that perhaps Ld IL-2 can also balance the immune disorders of D2T RA, particularly the RA subtype. However, evidence on the clinical efficacy and safety of low-dose IL-2 in the treatment of D2T RA is still lacking.

In this retrospective study, we delved into the lymphocyte subsets and CD4^+^ T-cell subsets using modified flow cytometry to explore the uniqueness of D2T RA. All RA patients received DMARDs with or without low-dose IL-2 to further clarify the clinical feasibility of IL-2 therapy for D2T RA.

## 2 Materials and methods

### 2.1 Participants

The participants included 1,042 patients with RA and 339 HCs. All patients were admitted to the Rheumatology Department of the Second Hospital of Shanxi Medical University from June 2019 to September 2020, who met the 1987 and 2010 RA classification criteria (20, 21). The HCs were healthy people recruited from the physical examination center of the hospital. Patients with RA (a total of 1,042) were divided into three groups: 239 in the new RA group, 478 in the treated RA group, and 325 in the D2T RA group.

New RA is defined as newly diagnosed RA patients who have never taken hormones and DMARDs. Treated RA is defined as patients whose disease is in remission when given hormones and one to two biologic agents or DMARD-targeting drugs. D2T RA refers to patients who are given at least two biologic agents or DMARD-targeting drugs, the course of treatment being more than half a year, and the disease is still repeated, showing a progressive worsening trend. All subjects have the right to informed consent and sign the informed consent form. The study was approved by the Ethics Committee of the Second Hospital of Shanxi Medical University [approval no (2019). YX No (105).].

The exclusion criteria included the co-occurrence of other autoimmune diseases and other diseases that may have affected the results, such as tumors or severe infections, as well as being pregnant and nursing women and children under 18 years of age.

### 2.2 Treatment

A total of 381 patients with RA (consisting of 107 in the new RA group, 151 in the treated RA group, and 123 in the D2T RA group) received additional low-dose IL-2 treatment (0.5 MIU/day was injected subcutaneously for five consecutive days) in combination with conventional immunosuppressive therapy. The most common adverse reactions were injection site reactions, fever, and influenza-like symptoms. Patients were closely monitored for vital signs, respiratory discomfort, and injection site reactions within 2 weeks of subcutaneous IL-2 injection. The absolute number of Treg cells in these RA patients treated with low-dose IL-2 was below the normal range.

### 2.3 Clinical and laboratory data collection

The demographic and laboratory data of the RA patients were recorded, including white blood cell (WBC) count; red blood cell (RBC) count; platelet (PLT) count; lymphocyte (LY) count; erythrocyte sedimentation rate (ESR); C-reactive protein (CRP); Disease Activity Score-28 (DAS28); levels of rheumatoid factor (RF), anti-cyclic citrullinated peptide antibody (anti-CCP), and anti-mutated citrullinated vimentin (anti-MCV); and liver and kidney function tests. All laboratory tests were performed on freshly drawn blood from patients after fasting in the morning. Patients treated with low-dose IL-2 were re-evaluated with laboratory data within 2 days of treatment.

#### 2.3.1 Flow cytometry

The flow cytometer was set up via the BD FACSDiva™ CS&T Research Beads, and quality control was achieved through the BD Multi-Check™ Control. Flow cytometry was compensated by BD FACS™ 7-color Setup Beads.

##### 2.3.1.1 Determination of absolute counts of lymphocyte subsets in the peripheral blood

Peripheral blood lymphocyte subsets include total T lymphocytes, total B lymphocytes, natural killer (NK) cells, CD4^+^ T cells, and CD8^+^ T cells.

**Figure 1:**
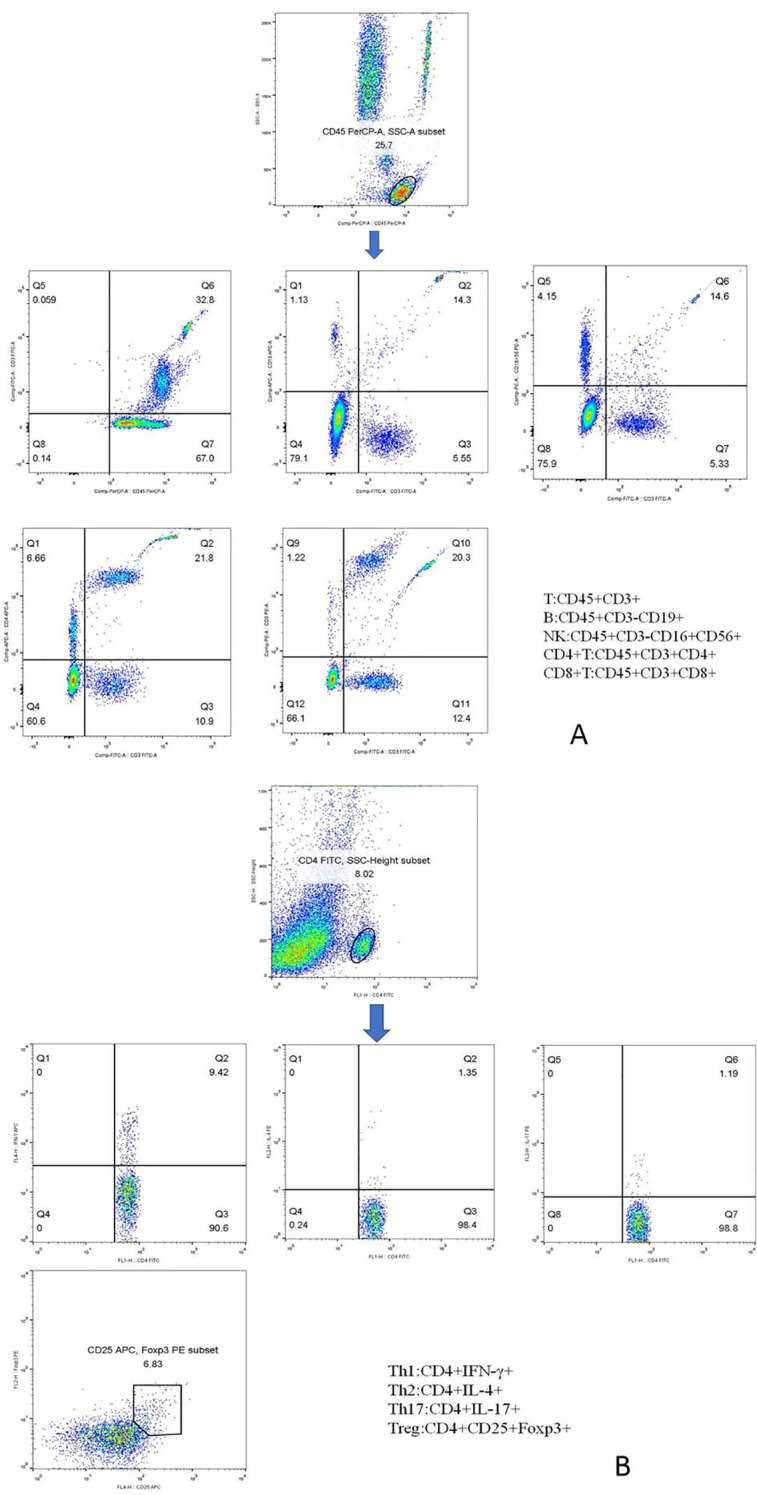
Phenotypic characterization of lymphocyte subpopulations. **(A)** Representative flow cytometry analysis of peripheral lymphocytes, including total T, total B, NK, CD4^+^ T, and CD8^+^ T cells. **(B)** Representative flow cytometry analysis of CD4^+^ T-cell subsets, including Th1, Th2, Th17, and Treg cells.


The specific method was as follows: two flow tubes containing a known number of fluorescent microspheres were labeled A and B, and 50 μL of peripheral blood was added using the reverse sampling method. The anti-CD3FITC/CD8PE/CD45PercP/CD4APC antibody and the CD3FITC/CD16 + 56-PE/CD45PercP/CD19APC antibody were added 20 μL each in the two tubes, respectively. The mixture was shaken well and incubated at room temperature away from light for 20 min. Then, 450 μL of XFACS hemolysin was added and incubated under the same conditions for 15 min. Each sample collected a total of 15,000 cells (**Figure 1A**). The principle is that the total lymphocyte count was calculated using a commercial reagent from BD. A certain amount of approximately 50,000 microspheres (the specific number will be indicated on the box) was placed in advance in the absolute counting tube, and 50 μL of whole blood was added. In this way, the ratio of blood and microspheres in whole blood cells was certain, the number of lymphocytes in 50 μL of whole blood can be calculated, and then the number of lymphocytes per microliter can be calculated. The specific formula is as follows:

$$\text{Lymphocyte counts = }\frac{\text{Number of cells obtained by FCM }(\text{known})}{\text{Number of beads obtained by FCM }(\text{known})}\times \text{Absolute number of beads in the counter tube}$$

$$\text{Cell count}/\text{μ}\text{L}=\frac{\text{Lymphocyte counts}}{50\;\text{μ}\text{L}}$$

##### 2.3.1.2 Determination of absolute counts of CD4^+^ T-cell subsets in the peripheral blood

Peripheral blood CD4^+^ T-cell subsets included Th1 cells, Th2 cells, Th17 cells, and Treg cells. The specific methods were as follows: 10 μL of phorbol myristate acetate working solution, 10 μL of ionomycin working solution, and 1 μL of GolgiStop were added to 80 μL of peripheral blood samples and incubated at 37°C in an incubator containing 5% CO_2_ for 5 h. Anti-CD4-FITC was added and stained at room temperature away from light. One milliliter of freshly prepared fixing/penetrant solution was mixed and incubated at 4°C for 30 min away from light. Th1 cells were detected with anti-FITC-CD4 and anti-IFN-γ-APC, Th2 cells with anti-FITC-CD4 and anti-IL-4-PE, and Th17 cells with anti-human IL-17-PE. Treg cells were added with the CD4-FITC antibody and CD25-APC antibody in 80 μL of blood samples, incubated at room temperature for 30 min away from light, and added with 1 mL of freshly prepared fixed/permeable mixture solution, and anti-FOXP3-PE was detected under the same conditions. Finally, the cells were washed twice with a phosphate buffer. In the same way, we established the isotype controls. All immunofluorescence antibodies were purchased from BD Biosciences. All stained cells were assessed using flow cytometry (FACSCanto II; BD Biosciences, Franklin Lakes, New Jersey, USA). The data were analyzed using FlowJo V7.6.1 (Tree Star Inc., Ashland, OR, USA). The absolute number of cells in each subpopulation was calculated (percentage of counted cells × absolute number of CD4^+^ T lymphocytes). The markers of each CD4^+^ T-cell subpopulation are shown in **Figure 1B**.

#### 2.3.2 Cytometric bead array

Four milliliters of venous blood was left for 2 h, and the isolated serum was stored at −20°C. Cytometric bead array (CBA) was used to detect IL-2, IL-4, IL-6, IL-10, IL-17, IFN-γ, and TNF-α. The captured standard and test specimen data were transferred to the BD FCAP array software, and the test results were expressed in pg/mL.

### 2.4 Statistical analysis

The data conforming to the normal distribution were expressed as mean ± standard deviation (SD) and were analyzed using the independent sample *t*-test or ANOVA test. Data that were not normally distributed were expressed as the median (range) and were analyzed using the Mann–Whitney *U* test or the Kruskal–Wallis *H* test. Categorical variables were expressed in frequency and compared using chi-square tests. The paired sample *t*-test was used to analyze the changes of various data in RA patients after low-dose IL-2 treatment. A two-tailed *p*-value <0.05 was considered statistically significant.

## 3 Results

### 3.1 Clinical and demographic features

A total of 339 HCs (201 female and 138 male patients) with a mean age of 47.24 ± 13.27 years were enrolled. Demographic and clinical data for the three experimental groups (new RA, treated RA, and D2T RA groups) are shown in **Table 1**. There was no significant difference among the three groups regarding the indicators of disease activity, including ESR and CRP, and the indicators of RA-related autoantibodies, such as RF, anti-CCP, and anti-MCV. However, DAS28 scores in the D2T RA group were significantly higher than those in the new and treated RA groups. The platelet count in the treated RA group was higher than that in the new and D2T RA groups, but all three groups had platelet counts in the normal range. In terms of liver and kidney function, aspartate transaminase (AST) in the D2T RA group was higher than that in the new and treated RA groups. Compared with the new and D2T RA groups, alkaline phosphatase (ALP) in the treated group was higher, but blood urea nitrogen (BUN) was lower. There was no significant difference in drug use between the treated group and the D2T group, with 74.48% and 79.38% of patients using methotrexate (MTX), respectively, compared with 76.15% and 81.81% for leflunomide (LEF).

**Table 1:** Demographic and clinical characteristics.

	New RA (*n* = 239)	Treated RA (*n* = 478)	D2T RA (*n* = 325)
Demographics			
Age (years)	54.24 ± 14.13	56.95 ± 12.28	55.56 ± 13.31
Female, *n* (%)	160 (66.95%)	344 (71.97%)	226 (69.54%)
Disease duration (months)	121.00 (39.50–132.50)	126.00 (42.50–126.50)	132.00 (51.50–128.00)
Laboratory characteristics			
ESR (mm/h)	46.00 (25.00–100.00)	43.00 (24.00–83.00)	41.00 (26.00–71.00)
CRP (mg/mL)	18.35 (5.85–47.73)	15.40 (4.80–55.90)	14.90 (4.99–45.30)
DAS28	5.08 (3.81–6.16) ***	4.98 (3.90–6.17)***	5.71 (4.63–6.91)
RF (U/mL)	530.08 ± 226.96	524.10 ± 221.74	544.82 ± 218.13
Anti-CCP (U/mL)	1,144.84 ± 243.69	1,151.68 ± 244.09	1,132.03 ± 243.79
Anti-MCV (U/mL)	864.19 ± 156.18	871.51 ± 156.80	877.01 ± 160.66
ANA, *n* (%)	179 (74.90%)	318 (66.53%)	228 (70.15%)
WBC (*10^9^/L)	6.67 ± 2.53	7.05 ± 2.95	6.72 ± 2.79
RBC (*10^12^/L)	4.17 ± 0.58	4.18 ± 0.52	4.18 ± 0.73
PLT (*10^9^/L)	277.03 ± 104.28*	297.70 ± 108.37	268.43 ± 106.45***
LY (*10^9^/L)	1.57 ± 0.65	1.69 ± 0.72	2.29 ± 0.63
ALT (U/L)	21.72 ± 19.14	21.51 ± 26.41	20.25 ± 17.61
AST (U/L)	22.21 ± 13.27***	22.01 ± 19.85***	25.70 ± 42.55
ALP (U/L)	81.38 ± 50.82*	91.62 ± 48.47	75.20 ± 39.43**
GGT (U/L)	29.91 ± 40.42	30.41 ± 34.58	24.41 ± 30.56
BUN (mmol/L)	5.22 ± 1.94**	5.09 ± 1.83	6.02 ± 1.50***
CREA (μmol/L)	56.43 ± 25.00	55.37 ± 16.41	55.08 ± 32.62
UA (mmol/L)	259.44 ± 92.32	251.56 ± 78.26	265.22 ± 86.78
Medication			
Pred, *n* (%)	–	478 (100%)	325 (100%)
MTX, *n* (%)	–	356 (74.48%)	258 (79.38)
LEF, *n* (%)	–	364 (76.15%)	266 (81.85%)
Other DMARDs, *n* (%)	–	403 (84.31%)	291 (89.54%)

ESR, erythrocyte sedimentation rate; CRP, C-reactive protein; DAS28, Disease Activity Score-28; RF, rheumatoid factor; anti-CCP, anti-cyclic citrullinated peptide antibody; anti-MCV, anti-mutated citrullinated vimentin; ANA, antinuclear antibodies; WBC, white blood cell; Hb, hemoglobin; PLT, platelet; LY, lymphocyte; ALT, alanine transaminase; AST, aspartate transaminase; ALP, alkaline phosphatase; GGT, γ-glutamyl transpeptidase; BUN, blood urea nitrogen; CREA, creatinine; UA, uric acid; Pred, prednisone; MTX, methotrexate; LEF, leflunomide; DMARDs, disease-modifying antirheumatic drugs.

*p <0.05, **p <0.01, ***p <0.001.

### 3.2 The absolute number of CD4^+^ T cells and Treg cells decreased significantly in the D2T RA group

**Figure 2:**
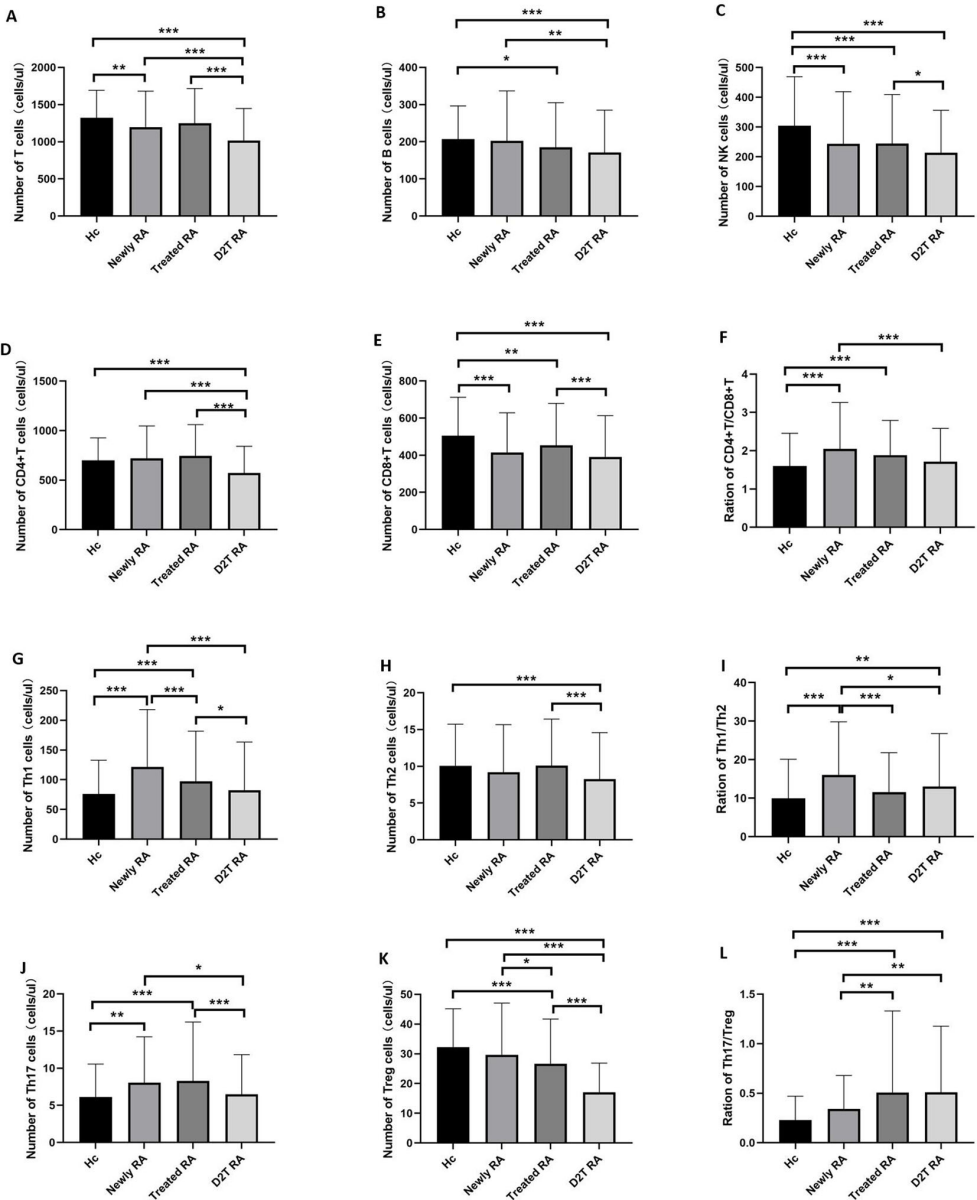
Peripheral T lymphocyte subpopulations in the new RA, treated RA, and D2T RA groups and HC. The absolute numbers of CD4^+^ T and Treg cells were significantly decreased in the D2T RA group. *p <0.05, **p <0.01, ***p <0.001. **(A–F)** represent the differences of T, B, NK, CD4+T, CD8+T cells and CD4+T/CD8+T cells in the three groups, respectively. **(G–L)** represent differences in CD4+T cell subsets among the three groups.

We compared the absolute number of lymphocyte subsets (including T, B, NK, CD^+^ T, and CD8^+^ T cells) and CD4^+^ T-cell subsets (including Th1, Th2, Th17, and Treg cells) in these four groups. The results are shown in **Figure 2** and **Supplementary Table S1**.

The first comparison was in terms of lymphocyte subsets. The number of T cells in the D2T RA group was significantly lower than that in the other three groups, and the number of T cells in the new RA group was lower than that in the HC group. Moreover, the number of B cells in the D2T RA group was lower than that in the HC and new RA groups, while that in the treated RA group was lower than that in the HC group. The results of NK cells and CD8^+^ T cells were similar, with the HC group having a higher number than the three experimental groups and the D2T RA group having a lower number than the treated RA group. In addition, the absolute number of CD4^+^ T cells in the D2T RA group was significantly lower than that in the other three groups. The ratio of CD4^+^ T/CD8^+^ T cells in the HC group was higher than that in the new and treated RA groups, and that in the D2T RA group was significantly lower than that in the new RA group.

Next was the comparison of CD4^+^ T-cell subsets. For Th1 cells, the new RA group had the highest number, followed by the treated RA group, and there was no statistical difference between the D2T RA and HC groups. Moreover, the Th2 cells in the D2T RA group were lower than those in the HC and treated RA groups. The number of Th17 cells in the treated RA group was the highest, followed by the new RA group, and there was no statistical difference between the D2T RA and HC groups. For Treg cells, of the highest concern to us, the D2T RA group had a significantly lower number than the other three groups; in addition, the treated RA group had a lower number than the HC and new RA groups. In terms of ratio, the ratio of Th1/Th2 and Th17/Treg cells in the D2T RA group was higher than that in the HC and new RA groups.

### 3.3 The cytokine levels in the new, treated, and D2T RA groups were significantly higher than those in the HC group

**Figure 3:**
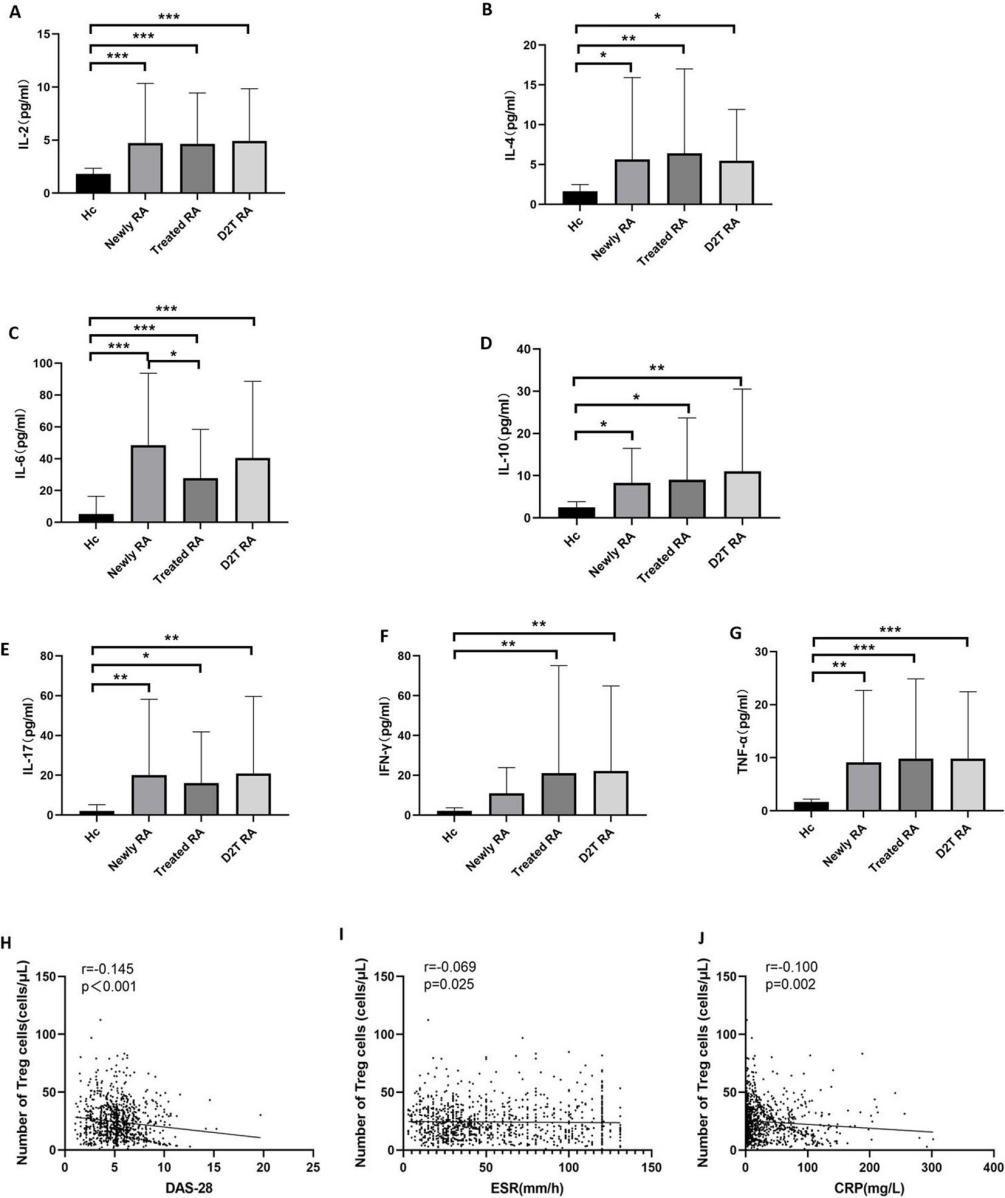
Cytokine levels and the correlation between Treg cells and disease activity. **(A–G)** Comparison of the cytokine levels in the new RA, treated RA, and D2T RA groups and the HC group. *p <0.05, **p <0.01, ***p <0.001. **(H–J)** Correlation between the absolute count of circulating Tregs and disease activity. The absolute count of Tregs in peripheral blood was negatively correlated with markers of disease activity, including ESR, CRP, and DAS28 in patients with RA. Significant values are asymptotic (two-sided tests) and the significance level is *p <*0.05.

We then compared the cytokine levels in the four groups, which can roughly represent immune cell function (**Figures 3A–G**, **Supplementary Table S2**). The levels of IL-2, IL-4, IL-6, IL-10, IL-17, and TNF-α in the three experimental groups were significantly higher than those in the HC group. Only the IFN-γ level of the new RA group was consistent with that of the HC group. In addition, the IL-6 level in the treated RA group was lower than that in the new RA group.

### 3.4 The number of Treg cells in RA patients was negatively correlated with DAS28 score, ESR, and CRP

Based on a large sample study, we analyzed the association between absolute Treg cell count and disease activity in patients with RA. The results showed that the number of Treg cells in RA patients was negatively correlated with DAS28, ESR, and CRP (*r* = −0.145, *p* < 0.001; *r* = −0.069, *p* = 0.025; and *r* = −0.100, *p* = 0.002, respectively) (**Figures 3H–J**).

### 3.5 Low-dose IL-2 treatment stimulated the proliferation of Treg cells in the new, treated, and D2T RA groups

A total of 381 RA patients were treated with low-dose IL-2, including new RA patients (66.39% female patients, mean age 56.16 ± 13.24 years), treated RA patients (68.21% female patients, mean age 55.37 ± 13.14 years), and D2T RA patients (66.67% female patients, mean age 56.83 ± 13.28 years). There was no difference in gender or age among the groups.

**Figure 4:**
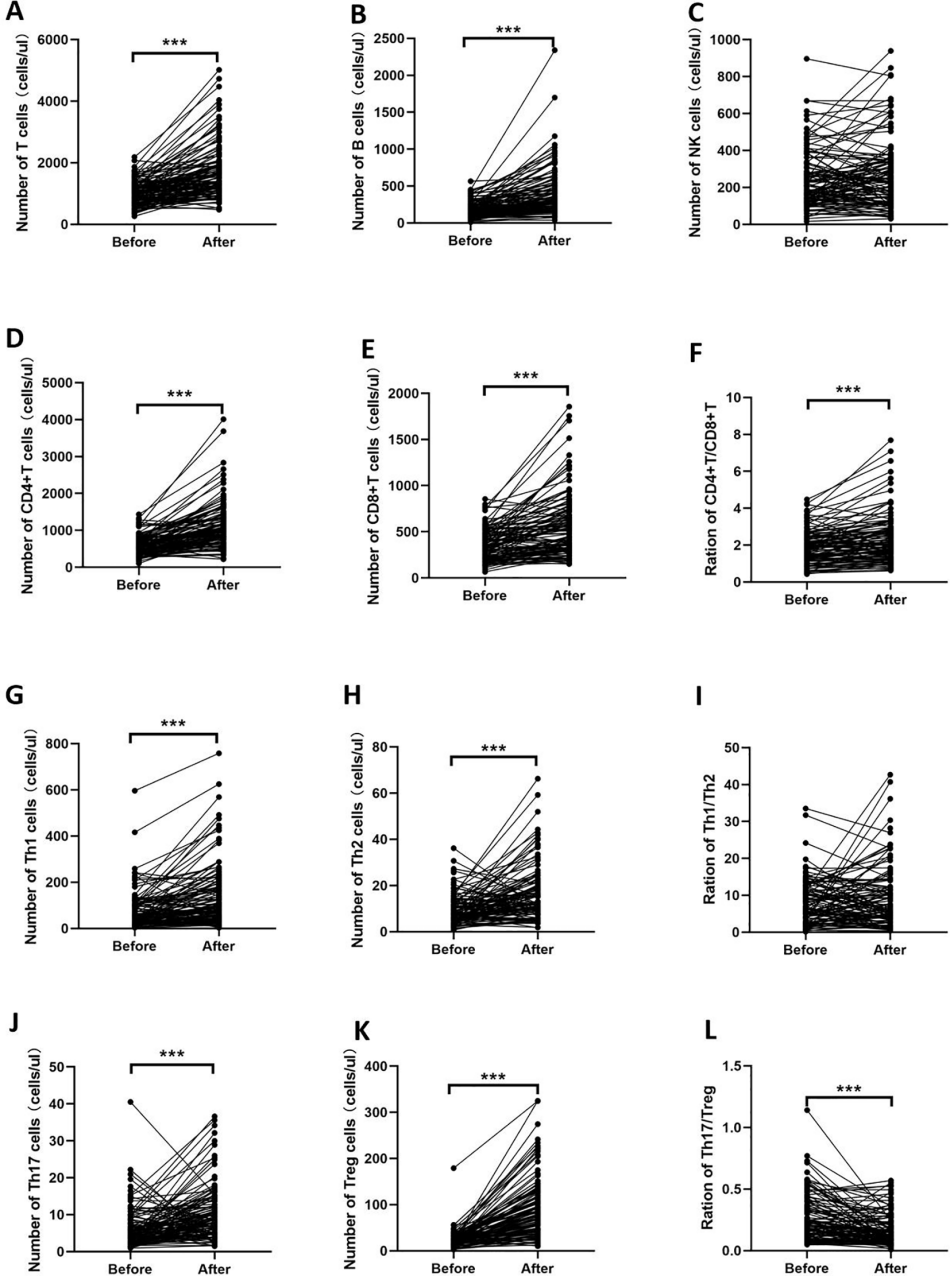
Changes in the number of lymphocyte subsets and CD4^+^ T-cell subsets of the D2T RA group. After low-dose IL-2 treatment, T, B, CD4^+^ T, CD8^+^ T, Th1, Th2, Th17, and Treg cells and the CD4^+^ T/CD8^+^ T-cell ratio increased, while the Th17/Treg cell ratio decreased significantly. **(A–F)** indicate the changes of T, B, NK, CD4+T cells, CD8+T cells and CD4+T/CD8+T cells before and after IL-2 treatment, respectively. **(G–L)** indicate the changes of CD4+T cell subsets before and after IL-2 treatment. ***p<0.001.

The absolute number of lymphocyte subsets and CD4^+^ T-cell subsets before and after low-dose IL-2 treatment in the three experimental groups is shown in **Supplementary Table S3**, **Supplementary Figures S1**, **S2**, and **Figure 4**. The results were similar in the three experimental groups. After low-dose IL-2 treatment, T, B, CD4^+^ T, CD8^+^ T, Th1, Th2, Th17, and Treg cells and the CD4^+^ T/CD8^+^ T-cell ratio increased, while the Th17/Treg cell ratio decreased significantly. In addition, there was no statistical difference in the ratio of NK cells and Th1/Th2 cells before and after treatment.

### 3.6 Disease activity decreased after low-dose IL-2 medication but did not affect liver and kidney function

Finally, we compared the changes in disease activity and some biochemical markers before and after treatment in all RA patients treated with low-dose IL-2. After low-dose IL-2 treatment, measures of disease activity such as ESR, CRP, and DAS28 were significantly reduced compared to baseline values (**Table 2**). ALT, AST, ALP, and GGT were used to evaluate liver function. AST and ALP decreased after treatment, but their levels remained within the normal range. The three indicators of kidney function, BUN, CREA, and UA, showed no significant change from baseline and were all within the normal range.

**Table 2:** Changes in disease activity indicators and liver and kidney function before and after low-dose IL-2 treatment.

	Before	After	*p*-value
Disease activity indicators			
ESR (mm/h)	70.18 ± 29.99	32.80 ± 16.60	<0.001***
CRP (mg/L)	51.79 ± 23.15	12.92 ± 5.82	<0.001***
DAS28	5.09 ± 0.64	3.59 ± 0.81	<0.001***
Liver function			
ALT (U/L)	28.60 ± 11.18	28.08 ± 9.48	0.324
AST (U/L)	27.12 ± 8.60	22.46 ± 7.51	<0.001***
ALP (U/L)	79.42 ± 22.83	75.95 ± 23.00	0.041*
GGT (U/L)	47.38 ± 21.74	48.51 ± 22.41	0.498
Kidney function			
BUN (mmol/L)	5.46 ± 1.46	5.47 ± 1.48	0.935
CREA (μmol/L)	60.01 ± 17.69	59.57 ± 17.96	0.734
UA (mmol/L)	321.06 ± 73.68	322.54 ± 73.52	0.777

ESR, erythrocyte sedimentation rate; CRP, C-reactive protein; DAS28, Disease Activity Score-28; AST, aspartate transaminase; ALP, alkaline phosphatase; GGT, γ-glutamyl transpeptidase; BUN, blood urea nitrogen; CREA, creatinine; UA, uric acid. *p<0.05, ***p<0.001.

Low-dose IL-2 was well tolerated in all RA patients without serious adverse reactions. Non-serious adverse events were characterized by a rash at the injection site that heals on its own without special treatment. There were also 12 patients with fever symptoms, but all of them returned to the normal range after physical cooling.

## 4 Discussion

In order to avoid immune responses against autoantigens, most autoreactive T cells are eliminated during thymus selection (22, 23). However, this selection process is “leaky,” which means that potentially harmful self-specific T cells with immune activity may escape elimination and be released from the thymus (24). For healthy individuals, these autoimmune T cells usually do not mediate the occurrence of disease due to the presence of negative regulatory cells such as Treg cells (25, 26), which is known as CD4^+^CD25^+^FOXP3^+^ T cells. This gives rise to the concept of an imbalance between Treg cells and effector T cells. Many studies have proven that for most autoimmune diseases, including RA, this imbalance is not due to the hyperactivity of effector T cells but the insufficiency of the number or function of Treg cells (27, 28).

At present, the results of several studies on peripheral blood Treg cell levels in RA are controversial (19, 29, 30), and most studies focus on exploring the differences in Treg cells in RA patients with different disease activity levels (19). When it comes to D2T RA, this heterogeneous RA subgroup has been particularly poorly studied. In 2023, a study (7) from the University of Tokyo, Japan, summarized the unique immunophenotype of D2T RA from the aspects of genes, autoantibodies, immune cells, type I interferon, etc. In terms of immune cells, B cells, T cells, monocytes, dendritic cells, neutral granular cells, and synovial fibroblasts were mainly studied, and the overall research scope was wide but slightly shallow. The present study investigated the level of peripheral Tregs in a large sample of RA patients. In this study, we explored the difference in cell number and function among D2T RA, healthy control, new RA, and treated RA at the level of lymphocytes and lymphocyte subsets. The results showed that the number of lymphocyte subsets including total T, total B, NK, CD4^+^ T, and CD8^+^ T cells and CD4^+^ T-cell subsets including Th1, Th2, Th17, and Treg cells in D2T RA patients was lower than that in the new, treated RA, and healthy control groups, while the IL-6, IL-17, and TNF-α cytokines were significantly higher than those in the healthy control group. This suggests that the number of lymphocytes in D2T RA patients is lower and more inhibited, but the function is in a hyperactive state. Perhaps, we think the reason is that Treg cells are reduced more, which causes the balance of effector T cells/Treg cells to shift in the direction of effector T cells, that is, autoimmune T cells become more aggressive against their own antigens. Therefore, the clinical manifestations of D2T RA patients are high severity, long clinical course, and poor treatment response.

Our study found that D2T RA patients had high DAS28 scores and decreased platelet levels, which were within the normal range. A common understanding is that platelet levels go hand in hand with disease activity, but our study found a contradictory result. However, this condition has not been reported in the literature, and there is no obvious bleeding tendency in D2T RA patients clinically. Our study also conducted a correlation analysis and found that Treg cell count in RA patients was negatively correlated with disease activity including DAS28, ESR, and CRP, which is similar to previous studies (31, 32).

The traditional view is that RA is mainly caused by over-strong immune function, so DMARDs and other drugs are used in clinical practice, mainly to inhibit or eliminate effector T cells (33). However, as mentioned above, the immune cells of D2T RA patients are in a low level and suppressed state, so the therapeutic effect of this “immunosuppressive means” on D2T RA is not ideal, and patients are prone to drug tolerance and relapse, and long-term use of DMARDs and other drugs will increase the risk of infection and tumor (1, 34, 35). At this point, low-dose IL-2 showed great therapeutic potential as an effective Treg cell stimulator.

The development of IL-2 for more than 50 years has proven that it is essential for thymus development and survival, development, and differentiation of Treg cells in peripheral lymphatic organs, and its relationship with immune tolerance has become increasingly clear (16, 24, 36). IL-2 is a 15.5-kDa four-alpha-helix bundle glycoprotein comprising 133 amino acids and is produced mostly by activated T cells and B cells (16). As a member of the common gamma-chain family of cytokines, IL-2 exerts its pleiotropic effects by binding its cell surface receptor complexes which are made up of three subunits: the IL-2 receptor α-chain (IL-2Rα; CD25), the IL-2 receptor β-chain (IL-2Rβ; CD122), and the common gamma-chain (IL-2Rγc; CD132). Many studies show that functional Tregs are highly dependent on IL-2 and their dominant suppression of effector T cell cells is lost upon removal of IL-2 signaling (37–39). Treg cells constitutively overexpress the IL-2 receptor complex (CD25, CD122, and CD132) (40, 41) and are therefore more sensitive to IL-2 than effector T cells, so only low doses of IL-2 are required to stimulate its expansion (24, 42). Signaling through the IL-2 receptor causes heterodimerization of the IL-2Rβ and γ-chain cytoplasmic domains, leading to the recruitment of Janus kinase (JAK) non-receptor tyrosine kinases such as JAK1 and JAK3. These JAK1 and JAK3 proteins phosphorylate tyrosine residues on the IL-2Rβ chain in turn and then propagate signal transduction through three pathways: in effector T cells, it is the Ras/Raf/MAPK and PI3K/Akt/mTOR pathways, while in Treg cells, it is the STAT5 pathway, which ultimately leads to the expression of the IL-2 target genes such as IL2RA, FoxP3, Cyclin D2, and Bcl-2 (43–45). Signaling through the PI3K/Akt/mTOR pathway is inhibited in Tregs through high expression of PTEN (phosphatase and tensin homolog), and this mechanism restricts Treg proliferation in response to IL-2. In contrast, effector T cells strongly proliferate in response to IL-2-mediated PI3K/Akt/mTOR signaling, and this response can be inhibited using the immunosuppressive drug rapamycin which targets mTOR to bring about cell cycle arrest.

In previous studies, the effectiveness of low-dose IL-2 on Treg cell proliferation and disease remission in psoriatic arthritis (PsA) (46), systemic lupus erythematosus (SLE) (47), and Sjögren’s syndrome (SS) (48) has been demonstrated, but side effects have not been evaluated. Another study on RA evaluated the side effects of low-dose IL-2 but did not group RA according to how difficult it was to treat (19). Our study demonstrated that low-dose IL-2 can significantly increase the number of Treg cells in the peripheral blood of patients with D2T RA and new and treated RA, but no difference in degree was observed. Moreover, we also concluded that low-dose IL-2 can significantly reduce disease activity in RA patients without additional liver and kidney burden and without significant side effects.

While we are aware that patients receiving different medications may have an impact on outcomes, these are not considered confounding factors. Patients with RA are generally treated with steroids and DMARDs, and the effects of glucocorticoids on Th subgroups, especially Th1 and Th17, have been studied and proven (49, 50). DMARDs were treated with low-dose IL-2, which further confirmed our finding that low doses of IL-2 stimulated Treg cell proliferation even though patients were also taking immunosuppressants.

Although this study included a large number of samples, long-term follow-up is needed to further confirm the efficacy and safety of low-dose IL-2 combination therapy. Second, as it was a retrospective study, peripheral blood samples did not suffice for *in-vitro* experiments. Third, the baseline medications in our RA patients differed, and neither ESR nor CRP was an accurate indicator of disease activity. Some of these issues may have had an important impact on our results.

In conclusion, the number of lymphocytes and subsets in D2T RA patients is low and inhibited, especially in Treg cells, but the function is hyperactive. Low-dose IL-2 can stimulate the proliferation of Treg cells in the peripheral blood of patients with D2T RA, shift the balance of effector T cells/Treg cells toward Treg cells, restore immune tolerance to a certain extent, and become a new means to improve the immune disorder of RA.

## Data Availability

The original contributions presented in the study are included in the article/**Supplementary Material**. Further inquiries can be directed to the corresponding author.
