# Supplementary material for: The absolute number of circulating Treg cells is reduced in difficult-to-treat RA patients and is ameliorated by low-dose IL-2
Source: Front Immunol. 2025 Feb 6;16:1522893. doi: 10.3389/fimmu.2025.1522893 (PMC11839615; doi:10.3389/fimmu.2025.1522893)
Supplement: Supplementary file 1 [file DataSheet1.docx]

Supplementary Material

# Supplementary Figures and Tables

## Supplementary Tables

| **Table S1:** Absolute lymphocyte and CD4+ T cell subset counts (cells/μL) | | | | | |
| --- | --- | --- | --- | --- | --- |
| Cell count  cells/μL | Newly RA  (n=239) | | Treated RA  (n=478) | D2T RA  (n=325) | Hc  (n=339) |
| Total T | 1120.33  (878.14-1480.48) | | 1192.96  (940.17-1552.00) | 936.00  (684.75-1293.71) | 1279.03  (1055.69-1569.39) |
| Total B | 167.38  (117.00-258.00)  () | | 157.00  (96.50-239.69) | 141.50  (85.62-217.25) | 191.00  (140.15-259.00) |
| NK | 205.21  (129.85-295.00) | | 206.29  (126.60-308.74) | 181.00  (105.65-280.70) | 272.88  (180.00-399.00) |
| CD4+T | 681.32  (474.00-896.22) | | 711.00  (539.49-910.34) | 535.94  (353.07-737.82) | 652.21  (544.22-827.38) |
| CD8+T | 373.64  (256.04-527.00) | | 412.00  (300.10-555.78) | 351.00  (236.00-497.95) | 460.22  (356.03-615.00) |
| Th1 | 101.32  (49.34-170.00) | | 77.35  (36.63-160.97) | 62.03  (25.45-107.04) | 72.17  (21.02-106.08) |
| Th2 | 7.74(4.93-11.87) | | 9.10(5.38-12.96) | 6.69(4.08-10.84) | 9.42(5.55-13.58) |
| Th17 | 6.74(3.77-11.00) | | 6.11(3.52-10.43) | 4.74(2.67-8.51) | 5.30(3.15-8.31) |
| Treg | 25.63  (17.07-39.02) | | 25.98  (15.41-35.00) | 15.50  (10.33-20.77) | 30.38  (22.87-41.26) |
| Th1/Th2 | 11.94(7.08-21.11) | | 8.82(4.30-15.47) | 9.43(4.22-16.56) | 7.50(2.26-14.25) |
| Th17/Treg | | 0.24(0.15-0.43) | 0.24(0.13-0.50) | 0.32(0.18-0.57) | 0.17(0.10-0.27) |
| Total T: total T cells; Total B: total B cells; NK: natural killer T cells; CD4+: CD4+ T cells; CD8+: CD8+ T cells; Th1:  T helper 1 cells; Th2: T helper 2 cells; Th17: T helper 17 cells; Treg: regulatory T cells; Th1/Th2: T helper 1 cell/ T helper 2 cell ratio; Th17/Treg: T helper 17 cell/regulatory T cell ratio. | | | | | |

| **Table S2:** Cytokine level | | | | | |
| --- | --- | --- | --- | --- | --- |
| Cytokine level(pg/ml) | | Newly RA  (n=239) | Treated RA  (n=478) | D2T RA  (n=325) | Hc  (n=339) |
| IL-2 | 4.72±5.62*** | | 6.30±15.90*** | 4.97±4.96*** | 1.80±0.55 |
| IL-4 | 5.65±10.27* | | 9.99±29.83** | 5.54±6.50* | 1.64±0.86 |
| IL-6 | 130.55±299.06*** | | 38.15±85.63*** | 76.37±236.45*** | 5.27±11.11 |
| IL-10 | 8.32±8.16* | | 8.99±14.52* | 11.12±19.70** | 2.51±1.35 |
| IL-17 | 20.01±38.19** | | 15.98±25.57* | 21.14±39.11** | 2.05±3.21 |
| IFN-γ | 9.64±12.46 | | 6.64±8.91** | 8.80±17.76** | 2.20±1.53 |
| TNF-α | 9.15±13.53** | | 23.28±116.47*** | 9.91±12.75*** | 1.67±0.55 |
| IL-2: Interleukin-2; IL-4: Interleukin-4; IL-6: Interleukin-6; IL-10: Interleukin-10; IL-17: Interleukin-17; IFN-γ: interferon-γ; TNF-α: Tumor necrosis factor-α.*P<0.05, **P<0.01,***P＜0.001. | | | | | |

| **Table S3**: Changes in the absolute number of lymphocyte and CD4+T cell subsets before and after low-dose IL-2 treatment | | | | | | |
| --- | --- | --- | --- | --- | --- | --- |
|  | Newly RA  (n=107) | | Treated RA  (n=151) | | D2T RA  (n=123) | |
| Cell count | Before | After | Before | After | Before | After |
| T | 1164.82±431.50 | 1880.72±900.89*** | 1043.33±428.06 | 1680.11±829.43*** | 1005.72±355.14 | 1739.61±889.26*** |
| B | 197.03±117.35 | 432.65±323.47*** | 181.19±118.35 | 395.77±339.30*** | 175.93±112.27 | 401.31±325.30*** |
| NK | 250.85±140.08 | 266.72±194.35 | 222.18±130.38 | 240.81±180.60 | 238.09±154.54 | 265.59±187.12 |
| CD4+T | 696.86±279.85 | 1195.58±605.07*** | 619.76±291.20 | 1078.77±596.24*** | 595.22±270.67 | 1110.29±613.91*** |
| CD8+T | 405.59±202.34 | 618.50±361.20*** | 368.88±189.93 | 539.94±306.22*** | 362.20±168.49 | 571.29±347.02*** |
| CD4+T/CD8+T | 1.98±0.96 | 2.25±1.15*** | 1.94±0.98 | 2.31±1.19*** | 1.87±0.94 | 2.29±1.23*** |
| Th1 | 76.22±63.64 | 138.07±145.47*** | 71.71±63.82 | 135.07±134.24*** | 65.65±61.05 | 129.24±124.69*** |
| Th2 | 11.01±7.76 | 17.10±13.05*** | 10.19±7.99 | 15.31±9.87*** | 9.80±7.58 | 16.64±13.35*** |
| Th1/Th2 | 8.86±8.31 | 9.59±9.21 | 8.90±8.03 | 10.03±9.00 | 8.24±7.48 | 9.47±8.71 |
| Th17 | 6.86±5.86 | 11.56±8.55*** | 6.28±5.49 | 10.80±8.37*** | 6.13±5.54 | 11.56±9.87*** |
| Treg | 36.02±16.94 | 106.98±69.69*** | 27.86±17.66 | 87.50±62.85*** | 24.00±13.22 | 90.89±64.73*** |
| Th17/Treg | 0.20±0.14 | 0.15±0.12** | 0.32±0.59 | 0.17±0.61*** | 0.35±0.65 | 0.17±0.13*** |
| RA:Rheumatoid arthritis; Before: before IL-2 medication; After: after IL-2 medication; T: T cells; B: B cells; NK: natural killer T cells; Th1: T helper 1 cells; Th2: T helper 2 cells; Th1/Th2: T helper 1 cell/r T helper 1 cell ratio; Th17: T helper 17 cells; Treg: regulatory T cells; Th17/Treg: T helper 17 cell/regulatory T cell ratio. | | | | | | |

## Supplementary Figures

**Figure legends:**

**Figure S1:** Changes in the absolute number of lymphocyte subsets and CD4+T cell subsets of Newly RA group.

**Figure S2:** Changes in the absolute number of lymphocyte subsets and CD4+T cell subsets of Treated RA group.


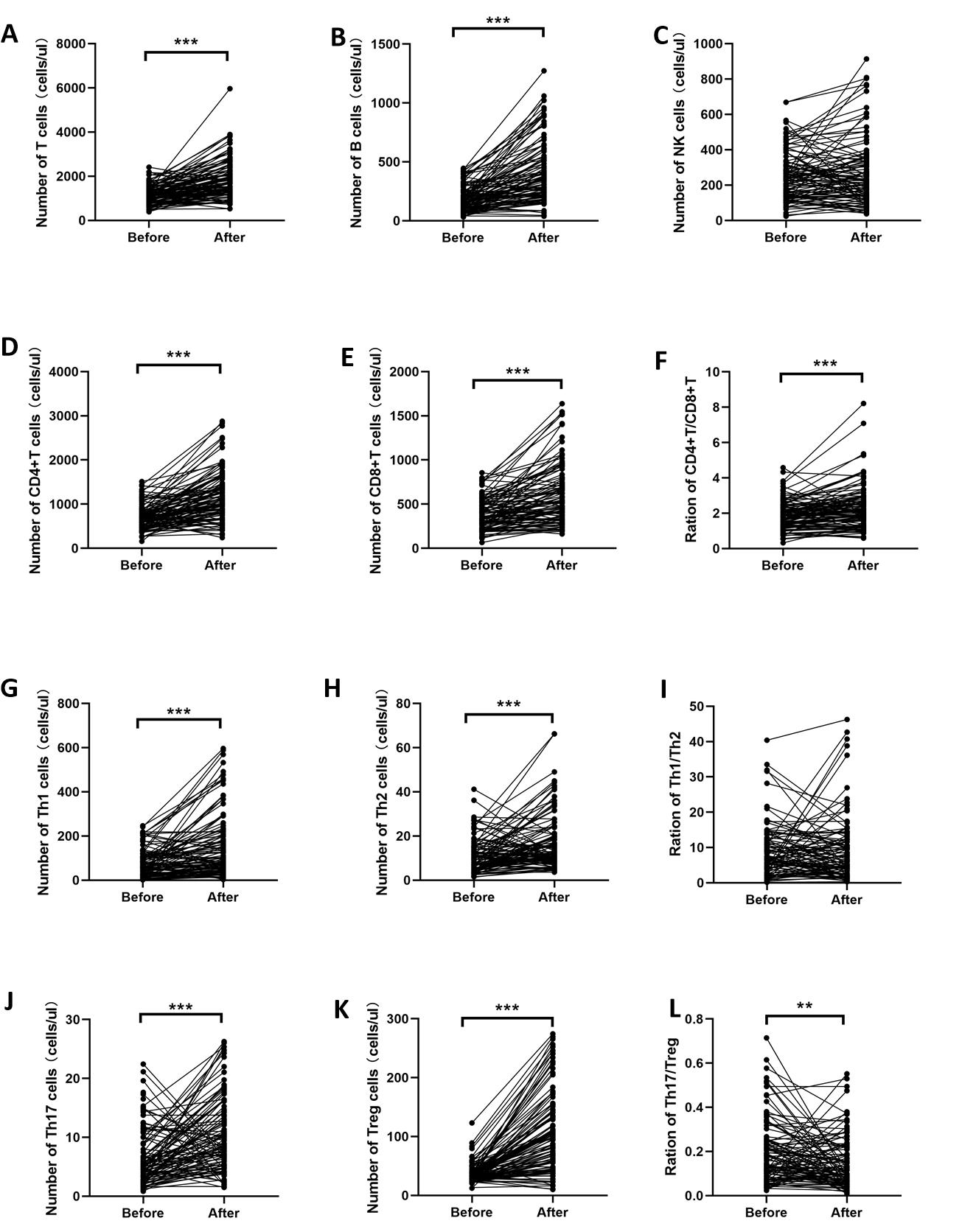


Figure 1


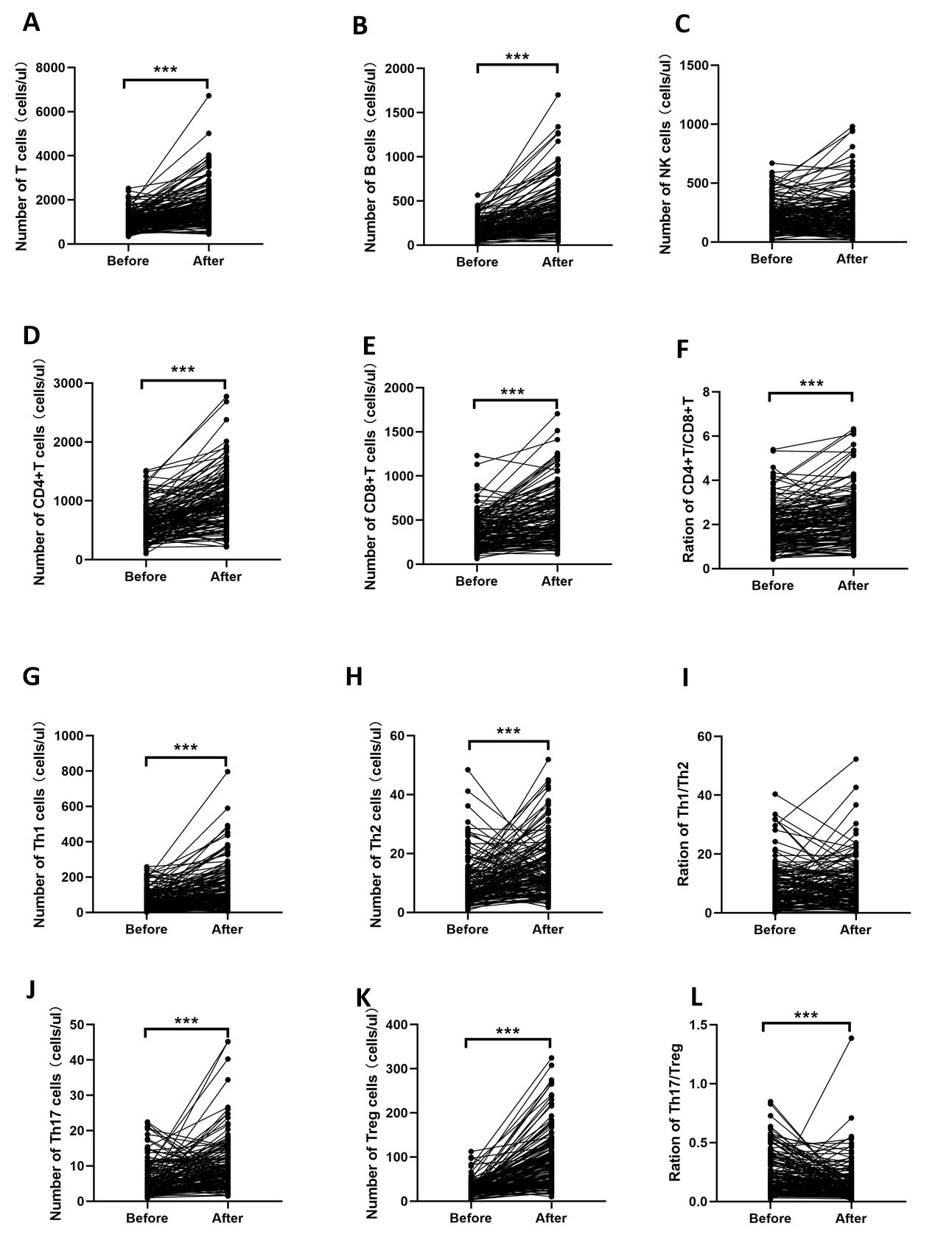


Figure 2
